# Graftless Immediate Dual Implant Anatomic Placement With Immediate Provisional Passive Loading and Definitive Hybrid Crown for the Restoration of Mandibular Molar: A Clinical Report

**DOI:** 10.7759/cureus.38654

**Published:** 2023-05-07

**Authors:** Hariharan Ramakrishnan, Praveen Sundar, Surabhi Halder, Shivakumar Baskaran, Mirza Rustum Baig

**Affiliations:** 1 Department of Prosthodontics and Implantology, Ragas Dental College and Hospital, Chennai, IND; 2 Department of Prosthodontics and Implantology, Priyadarshini Dental College and Hospital, Chennai, IND; 3 Department of Periodontics, Ragas Dental College and Hospital, Chennai, IND; 4 Department of Restorative Sciences, Faculty of Dentistry, Kuwait University, Jabriya, KWT

**Keywords:** endosseous dental implants, single tooth dental implant, immediate denture, immediate loading, immediate placement, immediate implant surgery, immediate dental implant loading

## Abstract

Immediate implant placement is well-known science and treatment in implant dentistry. It is a multitasking treatment consisting of surgical, prosthodontic, and periodontal aspects, implemented to obtain long-term clinically esthetic and functioning prosthesis. Immediate placement enables clinicians to reduce the number of surgical steps and shorter treatment duration. It has become a standard surgical protocol in modern implant practice. According to existing literature, dual implant placement can be done to avoid any cantilever effect in a single implant and to distribute masticatory forces.

This clinical report describes the extraction of an infected mandibular right first molar, (46, Federation Dentaire Internationale) followed by immediate dual placement of dental implants in the rinsed and cleansed sockets. The tooth was atraumatically extracted from the socket, and the latter was prepared to the required depth, and endosseous implants were placed in both the mesial and distal sockets. This atraumatic graft-free operating technique and immediate placement resulted in the preservation of hard and soft tissues. It also increased the patient's comfort, acceptance, and satisfaction due to immediate loading with a provisional removable prosthesis. This was later replaced with a dual screw-retained hybrid implant crown.

## Introduction

Immediate implants describe the placement of the implant into the extraction alveolus after the tooth extraction. Adequate patient selection and aseptic surgical methods should be followed [1]. Scientific data shows strong evidence that the clinical success rate of both the immediately placed implants and standard placement implants inserted in healed areas are almost similar [2].

The implant-supported prosthesis is considered a success when they clinically imitate adjacent natural teeth and surrounding harmonious soft tissue frame. Tooth loss leads to soft tissue collapse and bone resorption, resulting in a flat anatomical contour [3,4]. Therefore effective maintenance of existing soft and hard tissues must be esthetic goal management. One of the most common preservation methods of soft and hard tissues is the placement of immediate implants [5].

Immediately placed implants provide essential benefits such as less surgical intervention, shorter treatment time, and improved aesthetics. However, this approach requires a careful assessment of bone quality, amount of soft tissue, and biotype [6,7]. The essential prerequisites for a successful implant are primary stability during and after insertion implant loading. Evidence-based reports suggest that single implant placement has a better predictable success rate [8]. It had been stated that by following preoperative and post-operative protocols, immediate implants could be clinically placed into chronically infected sites [9].

## Case presentation

**Figure 1:**
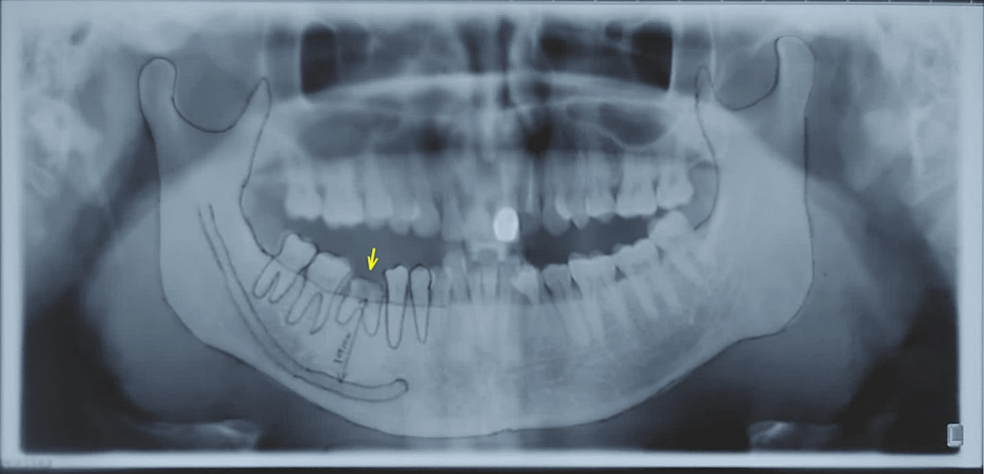
Preoperative orthopantomogram with the yellow arrow showing infected root stump in 46 regions (right mandibular first molar)

A 40-year-old male patient came to the Department of Prosthodontics and Implantology to enquire about treatment modalities for replacing missing natural teeth. There was no significant medical history. On intraoral examination, the patient reported a history of grossly decayed mandibular lower right molar. The patient was evaluated both clinically and radiographically. Radiographic evaluation revealed non-defined radiolucency involving enamel, dentin, and pulp, as evident in Figure 1.

No clinical or radiographic bone loss was noticed, so it was decided to place an implant immediately following extraction and curettage to avail the benefits of preservation of the bone. The patient desired to have a crown at the earliest. The treatment execution protocol was explained to the patient and was well accepted.

Surgical phase

**Figure 2:**
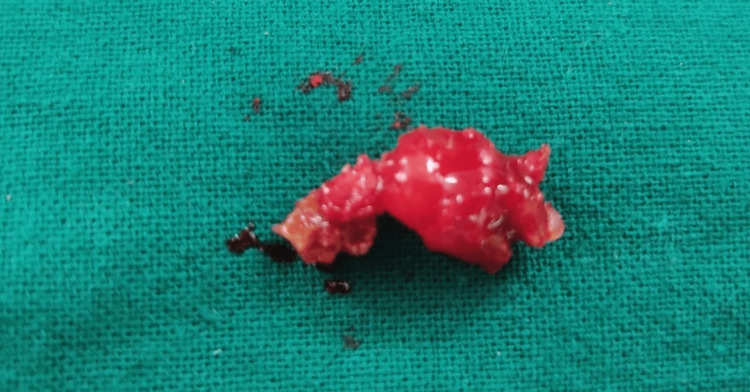
Extracted infected 46 (right mandibular first molar) root stump

The key to an immediate implant's success is atraumatic tooth extraction followed by curettage. This eventually leads to the preservation of alveolar bone. Local anesthesia was administered using lignocaine (Septodont Healthcare India Pvt. Ltd., Navi Mumbai, India; 1:80,000). Atraumatic tooth extraction was done using a periosteal elevator and periotomes (Figure 2).

**Figure 3:**
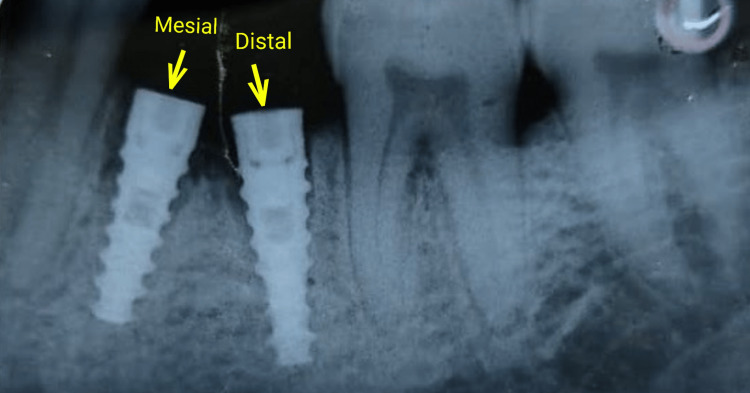
Implants with cover screws shown by yellow arrows placed in mesial and distal extraction sockets of 46 (right mandibular first molar)

The fragment was slowly luxated and extracted carefully without enlarging the socket excessively. Both the sockets were debrided with povidone-iodine (Amphray Labs, Mumbai, India), and two-part implants (Tuff, bone level; Noris Medical, Nesher, Israel) of dimensions 3.3mm x 11.5mm were placed individually following the immediate Implant placement protocol into the existing anatomy of the mesial and distal root sockets (Figure 3). Initially, pilot drill was utilised manually to gain purchase and alignment in socket and then the implants were placed individually. No other subsequent sequential drills were used as there was already sufficient implant bed following extraction.

**Figure 4:**
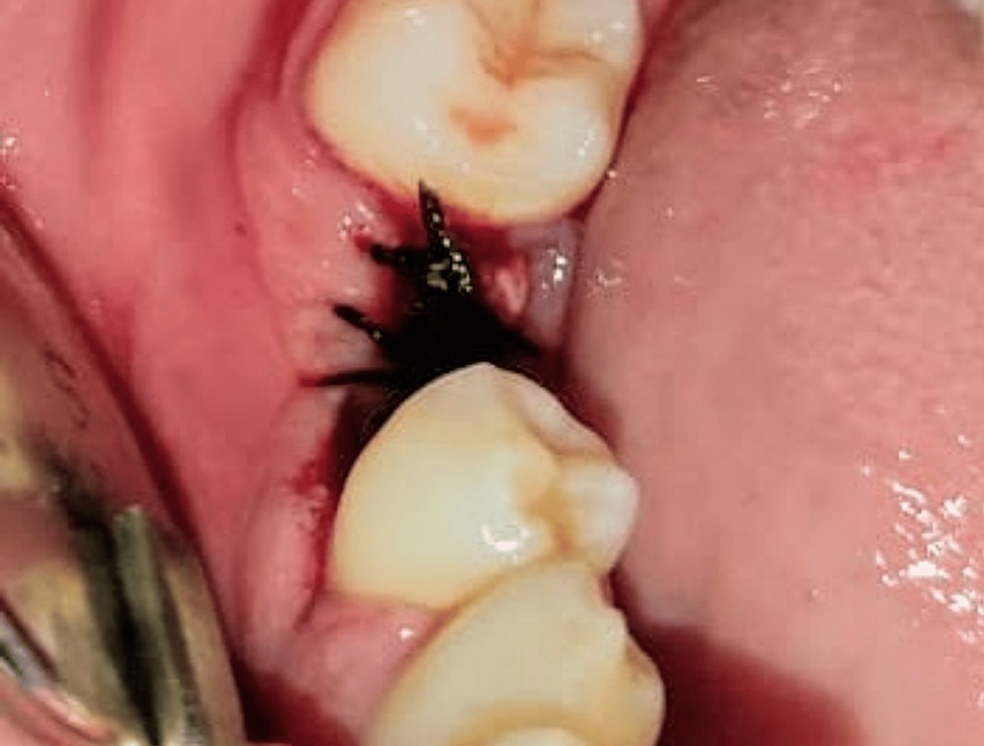
46 (right mandibular first molar) region sutured after placement of dual implants

No bone graft or connective tissue membrane was used during the entire process of dual implant placement. Cover screws were placed on both implants and interrupted 3-0 black braided silk sutures were placed (Figure 4).

Post-operative instructions were given to the patient, who was asked to report after a week. Oral rinse, antibiotics, and analgesics were prescribed for three days with a specific instruction to consume analgesics only when there is an increase in pain. Listerine mouthwash (Pfizer, New York, NY, USA), amoxycillin and potassium clavulanate tablets (Moxclav 625mg; SunPharma, Mumbai, India), paracetamol tablets (Dolo 650mg; Microlabs, Bangalore, India). The sutures were removed after a week. The patient was recalled after four months for final prosthodontic procedures.

Prosthodontic phase

**Figure 5:**
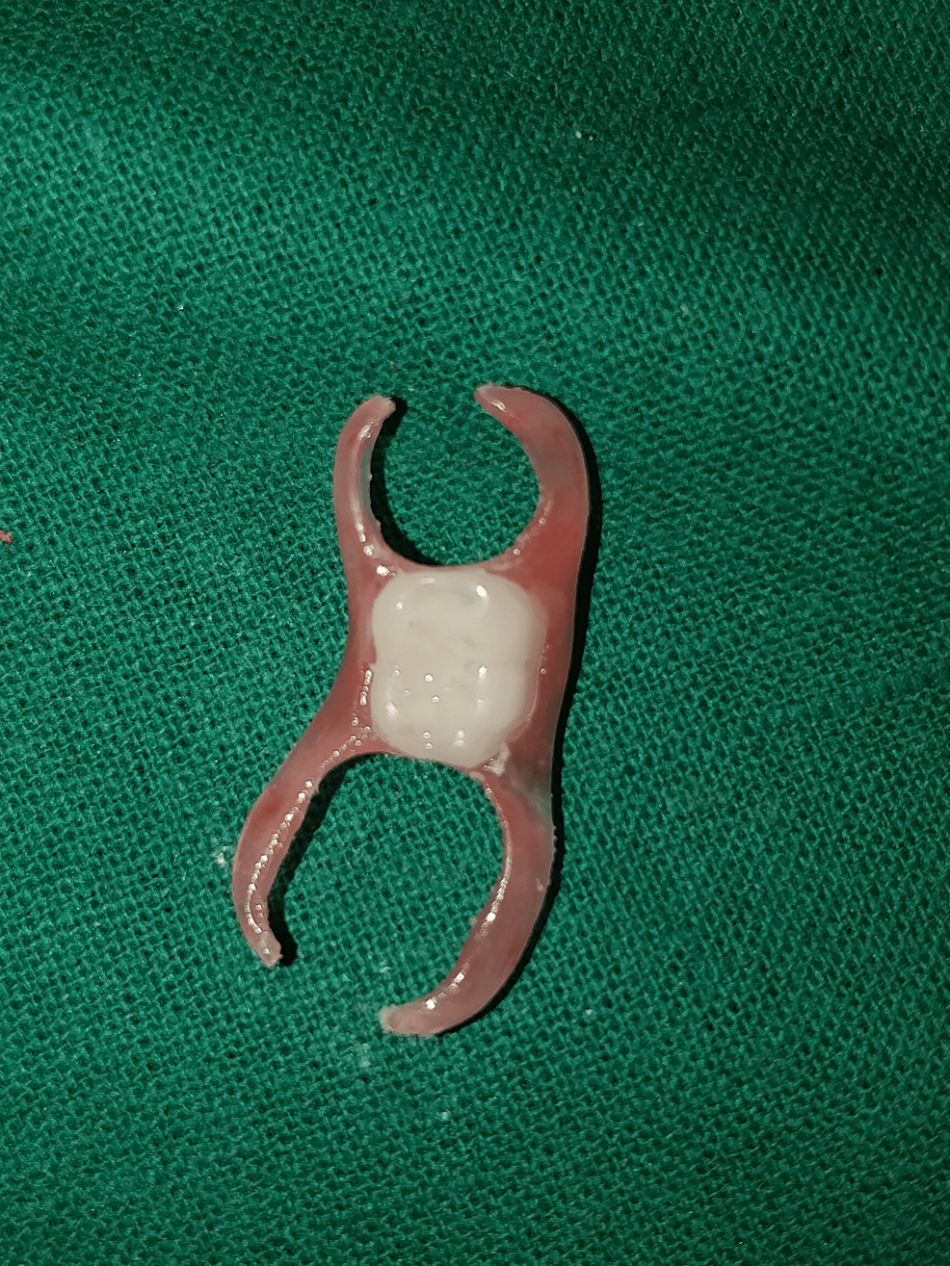
Flexible tooth-supported removable partial denture

**Figure 6:**
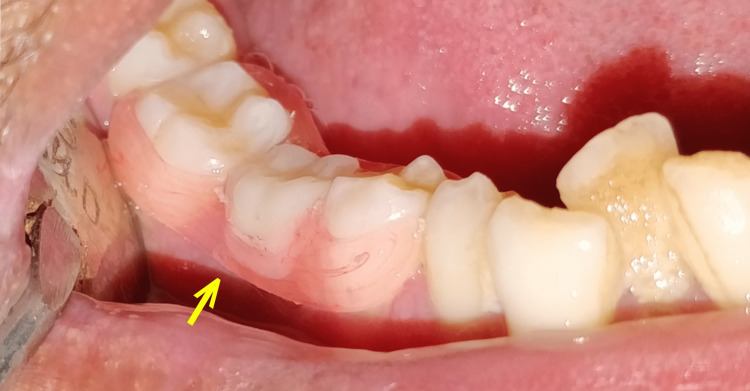
Yellow arrow showing well-placed buccal flange of removable partial denture

**Figure 7:**
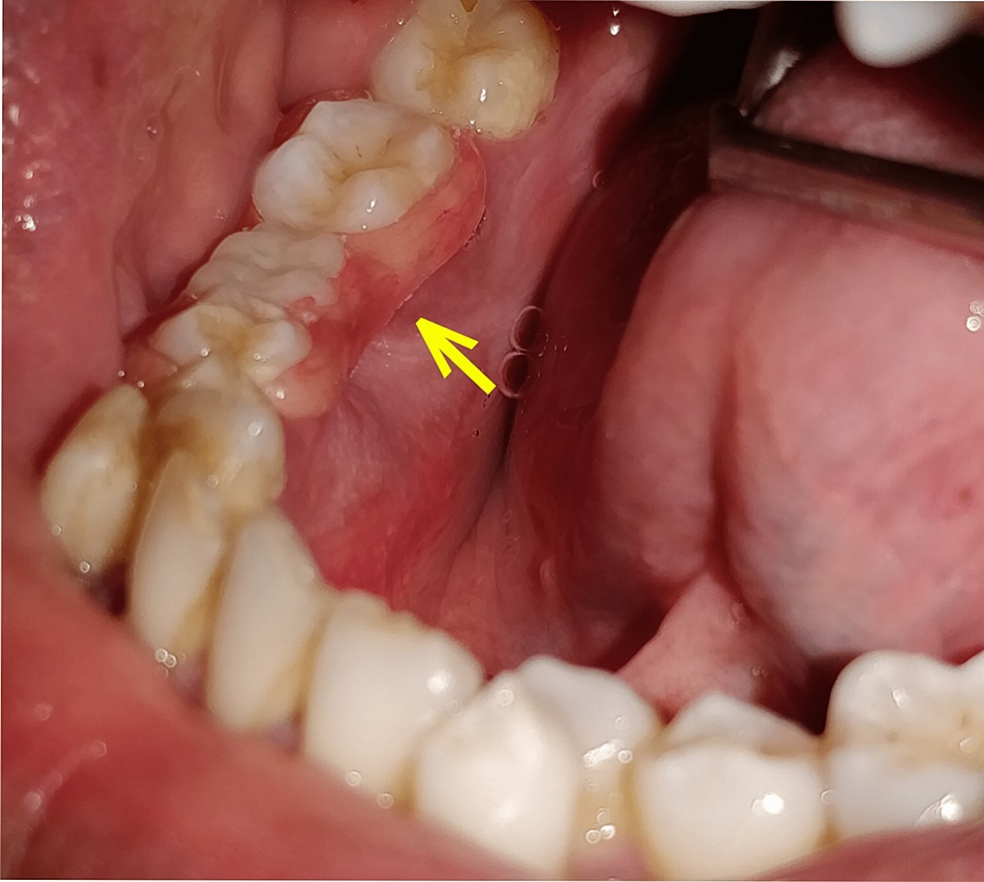
Yellow arrow showing well-placed lingual flange of removable partial denture

The primary stability of the mesial and distal implants followed good placement. Since the patient insisted on having an early tooth replacement, it was decided to passively load the sutured soft tissue area with a single tooth flexible, removable partial denture in implant protective occlusion with clasps (Figures 5, 6, 7).

**Figure 8:**
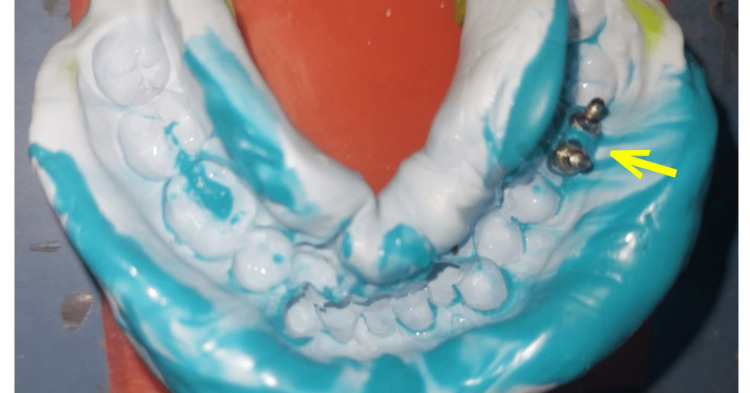
Putty and light body open tray implant impression with yellow arrow showing the diverged dual impression posts

On recall, after four months, the patient was evaluated intraorally and through an intraoral periapical radiograph (IOPA) for adequate osseointegration. The cover screws were exposed with a small incision, and circumferentially trimmed healing abutments were placed in both implants to have stable and screwable abutments (3mm; Noris Medical). Two weeks later, an adequate amount of gingival cuff was formed around the implants. The healing abutments were removed, and the open tray impression using carefully modified open tray implant impression copings (Noris Medical) was made using putty and light body (Aquasil addition silicone; Dentsply Sirona, Charlotte, NC, USA). Initially, the impression copings contacted each other due to a lack of parallelism in the dual implants. Therefore to enable a good impression, all the sides of dual copings were trimmed and altered to facilitate ease in impression making (Figure 8).

**Figure 9:**
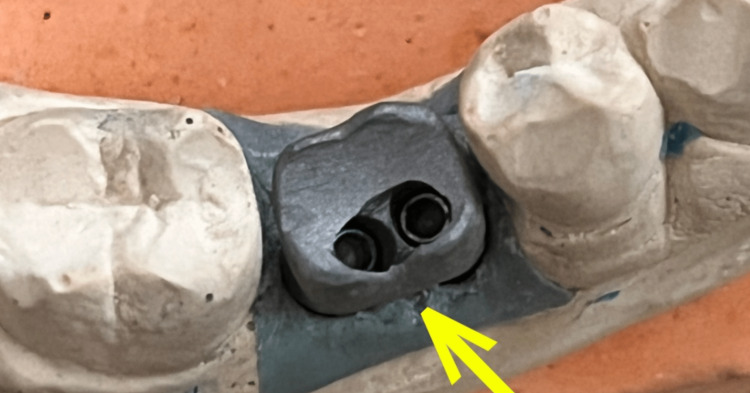
Arrow showing implant chrome cobalt crown with mesial screw access channel of castable abutment and distal screw access channel of 25-degree angulated titanium stock abutment

**Figure 10:**
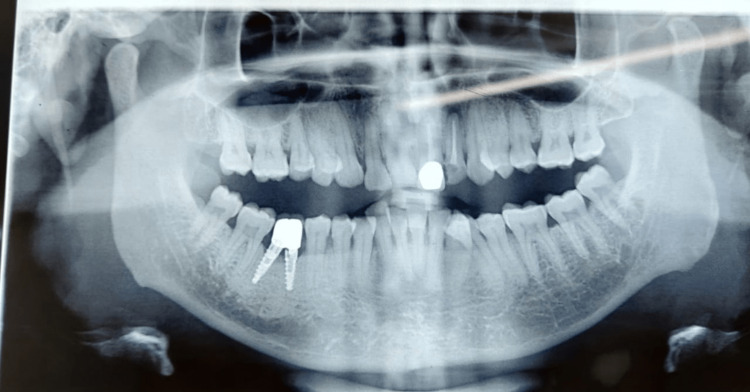
Postoperative orthopantomogram showing completed 46 (right mandibular first molar) dual screw-retained implant crown

**Figure 11:**
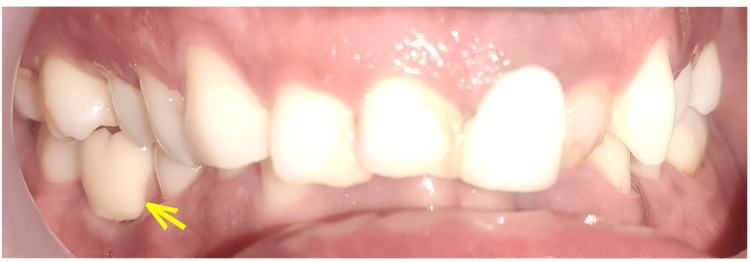
Intraoral picture with a yellow arrow showing the implant crown in implant protective occlusion

Healing abutments were again screwed back in position over two implants. A jig trial was performed to check for impression perfection. A bisque trial consisting of a porcelain fused to metal screw-retained implant crown made from cobalt chromium (castable abutment on mesial side; Noris Medical), and 30 degrees angulated titanium stock abutment (on the distal side; Noris Medical) was carried out (Figure 9). Later, the final implant crown was torqued in occlusion (Figures 10, 11).

**Figure 12:**
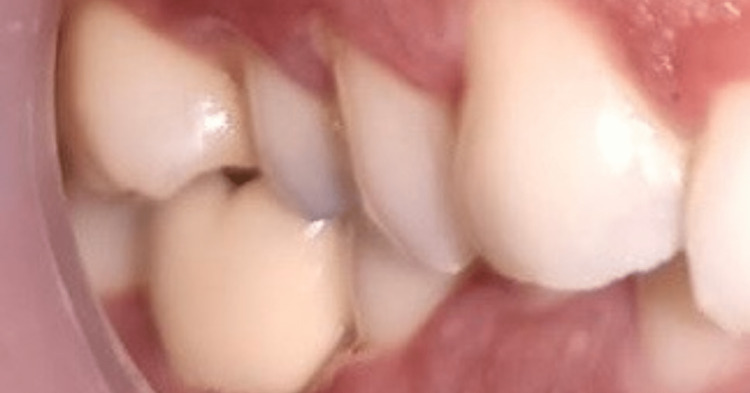
Buccal view of the implant crown (right first mandibular crown), after final torque

**Figure 13:**
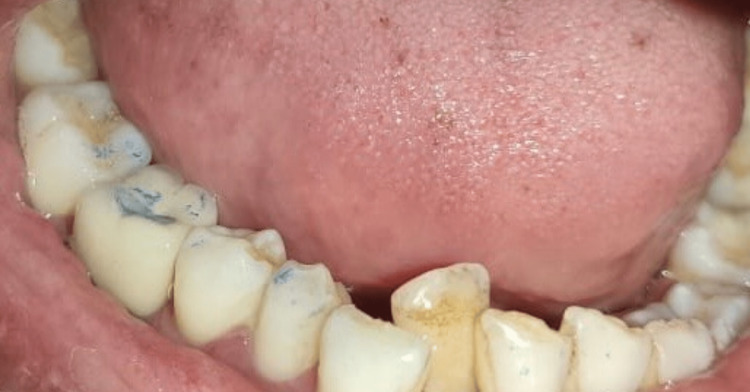
Occlusal view of first mandibular implant crown after final torque and during checking for occlusal errors

The masticatory surface of the crown had two openings (mesial and distal) and was adequately sealed with composite resin (Stedman, Chennai, India) without creating any high spots. Buccal and occlusal views of the implant crown immediately after final torque of 25 Ncm in both screws are shown in Figure 12 and Figure 13.

## Discussion

Many factors determine immediate implant placement in a fresh extraction socket. The initial stability of the implant is a primary factor in determining the success of implant placement [10]. To gain initial stability, the implant should be inserted a minimum of 3mm apical to the extraction alveolus site and 3mm apical to the available bone [11]. The significant advantage of immediate implant placement is reduced therapy time, surgical episodes, and preservation of gingiva and surrounding soft tissues. After tooth loss, a greater resorption rate occurs in bone during the first six months unless an implant is placed [12].

Immediate implant placement has high survival rate than implants inserted in non-extraction sites. One study states that the survival rate of implant insertion in the mandibular posterior region is comparatively less than that of the mandibular anterior region [13].

The titanium bases provide an alternative to avoid fracture in the connection of the implant abutment interface. The characteristic feature of titanium bases includes optimum resistance, adequate stability, and biocompatibility with adjacent soft tissues [14,15]. An implant with a diameter of 6mm in an 11.5mm mesiodistal edentulous area will have a 3mm cantilever causing shear stress acting directly on the bone and implant itself. Such a clinical scenario could lead to porcelain fracture, loosening of screws, fracture of implant, and increased bone loss. The ideal scenario is to get 1.5 plus 3.3 plus 3.0 plus 3.3 plus 1.5mm spacing. Here, 3.3mm is the diameter, 3.0mm is the distance between implants, and 1.5mm is between the natural tooth and the implant [15].

Previous studies stated that all implants inserted clinically were present at 10 to 18 months. Dual implant insertion has been successfully shown to be a more functional and esthetic method of molar replacement. Replacement of lost molar with dual narrow diameter dental implants is a promising surgery providing clinically feasible and predictable long-term positive clinical solutions [16,17]. Another study reported that using a diameter of 3.75mm implants in posterior regions resulted in up to 14% of implant loss. A dual implant insertion implant is clinically better than a single implant [18].

One study stated that regular‑diameter implants might not withstand occlusal chewing forces [19]. The surgical placement of two implants to replace a molar is an anatomic and scientific method to prevent complications. Lesser availability of the size of implants and their components in some systems is a moderate drawback. Immediate implant placement following tooth extraction was a predictable treatment for tooth loss [19,20].

## Conclusions

This clinical report described a minimally invasive graft-less surgical technique utilizing dual nonstandard diameter implants for replacing a molar by following the root anatomy while placing two implants instead of parallel placements, enhancing primary stability for immediate passive loading. Difficulties encountered during the prosthetic phase were clinically corrected to enable final prosthesis placement. Lesser chairside time and surrounding soft tissue area preservation added advantages to this technique. Proper patient selection, strategizing, and applying correct treatment, coupled with good post-surgical care, are vital for the clinical success of immediate implants placed without grafts.
